# Molecular Mechanisms Underlying Insect Behaviors: Receptors, Peptides, and Biosynthetic Pathways

**DOI:** 10.3389/fendo.2013.00120

**Published:** 2013-09-12

**Authors:** J. Joe Hull, Shogo Matsumoto

**Affiliations:** ^1^USDA-ARS, US Arid Land Agricultural Research Center, Maricopa, AZ, USA; ^2^Molecular Entomology Laboratory, RIKEN Advanced Science Institute, Wako, Japan

**Keywords:** intracellular signaling, neuropeptide, receptor, biosynthetic pathway, insect

Intracellular signaling pathways govern nearly every facet of insect life. As a consequence, significant effort has been made to expand our understanding (for both basic and applied purposes) of these pathways at the molecular and cellular levels. It is the aim of this Research Topic to highlight recent developments in insect systems that address fundamental questions of how chemical signals trigger biological/physiological responses. The articles presented here have been loosely grouped into three topics: receptors, ligands, and intracellular pathways/mechanisms.

The first section (four Original Articles and one Review Article) is “receptor-centric” with four papers discussing G protein-coupled receptors (GPCRs) and the other reporting on an intracellular nuclear receptor. Hypertrehalosemic hormone (HTH) is a peptide hormone recently implicated in some systems in the cellular response to oxidative stress. Huang et al. (1) report identification of a GPCR for HTH in the German cockroach (*Blattella germanica*). Based on RNAi-mediated knockdown of the ligand-receptor pair, the authors conclude that HTH and its receptor are critical for mediating anti-oxidative responses in *B. germanica*. Lee et al. (2) examined the expression of a different peptide hormone receptor, pheromone biosynthesis activating neuropeptide receptor (PBANR). They showed that four alternatively spliced variants, differentiated only by the length of their carboxyl terminal tails, are concomitantly expressed in the pheromone glands of multiple moth species. This finding addresses concerns regarding the biological significance of PBANR isoforms identified previously. To further evaluate the functionality and regulation of those PBANR variants, Lee et al. (3) established cultured insect lines constitutively expressing the receptor variants. Functional characterization of the receptors revealed that the “long” and “short” variants utilize different downstream effectors and regulatory mechanisms. Departing from the Original Article format of the previous entries, Bendena et al. (4) present a short review of select neuropeptides [NPF/Y, short neuropeptide F (sNPF), FMRFamide, pigment dispersing hormone, cholecystokinin, and allatostatin-like peptides] and their GPCRs in *Drosophila melanogaster* and *Caenorhabditis elegans*. The final paper in the receptor grouping used confocal scanning laser-based immunohistochemical imaging to examine nuclear trafficking of the ecdysteroid receptor (EcR) in *Rhodnius prolixus* cells (5). Co-localization of EcR with microtubules, Hsp90, FKBP52, and a component of dynein, along with impeded EcR nuclear localization following disruption of the microtubule network, led the authors to propose that EcR translocates to the nucleus along microtubules via a cytosolic dependent protein complex.

The second section (two Original Articles and one Review Article) of this issue focuses on neuropeptide ligands, in particular pheromone biosynthesis activating neuropeptide (PBAN) and sNPF. In most species of moths, PBAN controls biosynthesis of the chemical compounds (i.e., sex pheromones) that females use to attract conspecific males. To develop a more nuanced understanding of how PBAN interacts with its receptor [i.e., PBANR; see (2, 3)], Kawai et al. (6) sought to clarify the role of the Arg residue in the FxPRLamide active core of PBAN. Structure-function analyses revealed the importance of this residue in PBANR binding and activation. PBAN/pyrokinin peptides, which are characterized by the FxPRLamide motif, are present in multiple insect orders. In their Review Article, Choi and Vander Meer (7) discuss recent research on the molecular structure and diversity of PBAN/pyrokinin peptides in ants, in particular the fire ant, *Solenopsis invicta*. The final paper of this section examines the role of sNPFs in governing feeding behavior in the silkworm, *Bombyx mori* (8). MALDI-TOF mass spectrometric profiling revealed that brain sNPF levels decreased following starvation but returned to basal levels after feeding, while direct injection of sNPFs into larvae stimulated feeding.

The final section (two Review Articles, three Original Articles, one Mini-Review, and one Perspective) of this issue centers on a number of diverse intracellular pathways/mechanisms. The opening quartet of articles in this section focuses on the intracellular pathways associated with moth sex pheromone biosynthesis. The first article by Jurenka and Rafaeli (9) provides an extensive review of the role PBAN has in heliothine moth biosynthetic pathways, and discusses recent findings implicating PBAN involvement in male pheromone production. Ohnishi et al. (10) present an Original Article describing their use of *B. mori* EST databases in conjunction with RNAi screening to identify transcripts involved in *B. mori* sex pheromone production. Subsequent characterization of one such transcript identified an acyl carrier protein essential for biosynthesis of the *B. mori* sex pheromone precursor. In an Original Article, Moto and Matsumoto (11) address the limitation of unequivocally demonstrating the *in vivo* functionality of genes linked to sex pheromone biosynthesis in systems such as moths that lack the desired genetic malleability. They describe the development of a transgenic system in *B. mori* utilizing the piggyBac transposon in conjunction with a pheromone gland-specific desaturase promoter region. In the final article of the quartet, Watanabe et al. (12) present chemical evidence for the biosynthetic pathway utilized by the female rice looper (*Plusia festucae*) to generate ω7-unsaturated sex pheromone components. Intriguingly, comparative analysis with another Plusiinae species suggested that different β-oxidation systems are utilized. The Review Article by Stanley and Kim (13) moves the focus away from moth sex pheromone production and places it on the biological role of prostaglandin receptor-mediated events in insect physiology. Among the topics reviewed are the role of prostaglandins in egg-laying in crickets and insect egg development in general, a prostaglandin receptor in tick salivary glands and prostaglandin receptor-mediated action in insect immunity. In a Mini-Review Article, Tanaka (14) describes recent studies on the regulatory mechanism underlying ecdysteroidogenesis (i.e., the production of compounds critical to insect molting and metamorphosis). The author further highlights studies in *B. mori* that have examined the role of humoral factors and neural control on ecdysteroidogenesis. Negri (15) presents the last paper in this issue, a Perspective Article reviewing data implicating the involvement of *Wolbachia*, a widespread endosymbiont of arthropods, in modulating host insulin and ecdysteroid hormonal pathways. The author concludes by presenting a model illustrating a possible mechanism for direct bacterial influence on host ecdysone signaling and epigenetic regulation.
